# Purification and Characterization of a Novel Endolytic Alginate Lyase from *Microbulbifer* sp. SH-1 and Its Agricultural Application

**DOI:** 10.3390/md18040184

**Published:** 2020-03-31

**Authors:** Jin Yang, Dandan Cui, Diwen Chen, Wenkang Chen, Shuo Ma, Hong Shen

**Affiliations:** 1 College of Natural Resources and Environment, South China Agricultural University, Guangzhou 510642, China; jyangscau@126.com (J.Y.); cuidan0627@163.com (D.C.); chendiwen@126.com (D.C.); hnnd2017@163.com (W.C.); shuoma1994@163.com (S.M.); 2 Guangdong Bioengineering Institute (Guangzhou Sugarcane Industry Research Institute)/Guangdong Provincial Key Lab of Sugarcane Improvement & Biorefinery, Guangzhou 510316, China; 3 Guangdong Provincial Key Laboratory of Eco-Circular Agriculture, Guangzhou 510642, China

**Keywords:** alginate lyase, *Microbulbifer* sp. SH-1, purification, endolytic, alginate oligosaccharides, chilling stress

## Abstract

Alginate, an important acidic polysaccharide in marine multicellular algae, has attracted attention as a promising biomass resource for the production of medical and agricultural chemicals. Alginate lyase is critical for saccharification and utilization of alginate. Discovering appropriate and efficient enzymes for depolymerizing alginate into fermentable fractions plays a vital role in alginate commercial exploitation. Herein, a unique alginate lyase, AlgSH7, belonging to polysaccharide lyase 7 family is purified and characterized from an alginate-utilizing bacterium *Microbulbifer* sp. SH-1. The purified AlgSH7 shows a specific activity of 12,908.26 U/mg, and its molecular weight is approximately 66.4 kDa. The optimal temperature and pH of AlgSH7 are 40 °C and pH 9.0, respectively. The enzyme exhibits stability at temperatures below 30 °C and within an extensive pH range of 5.0–9.0. Metal ions including Na^+^, K^+^, Al^3+^, and Fe^3+^ considerably enhance the activity of the enzyme. AlgSH7 displays a preference for poly-mannuronic acid (polyM) and a very low activity towards poly-guluronic acid (polyG). TLC and ESI-MS analysis indicated that the enzymatic hydrolysates mainly include disaccharides, trisaccharides, and tetrasaccharides. Noteworthy, the alginate oligosaccharides (AOS) prepared by AlgSH7 have an eliciting activity against chilling stress in Chinese flowering cabbage (*Brassica parachinensis* L.). These results suggest that AlgSH7 has a great potential to design an effective process for the production of alginate oligomers for agricultural applications.

## 1. Introduction

Alginate is the most abundant structural polysaccharide of brown macroalgae, composing 22%–44% of its dry weight, including *Laminaria japonica*, *Ascophyllum nodosum*, *Sargassum fusiforme*, and *Ecklonia maxima* [1]. Alginate is a linear block copolymer of two uronic monomers, β-d-mannuronic acid (M) and its C-5 epimer α-l-guluronic acid (G), arranged in homogenous (polyM, polyG) or heterogenous (polyMG) block-like patterns [2]. Owing to its properties of gelation, thickening, stability, and chelating metal ions, alginate has been widely used in food and beverages, paper and printing, biomaterials, and pharmaceutical industries [3,4]. In addition, as one of the most abundant complex marine polysaccharides and low-cost material, alginate has excellent potential as a carbon source for further deep processes, such as the production of alginate oligosaccharides (AOS) or bioethanol [5]. Among them, AOS, degradation products of alginate, exhibited many important bioactivities, such as antioxidant, neuroprotective, antibacterial, antitumor, and plant growth-promoting activities [6,7].

Alginate lyases degrade alginate via β-elimination of the 4-*O*-glycosidic bonds and produce a series of unsaturated oligosaccharides that contain double bonds at the non-reducing end [8]. Based on the substrate specificities, alginate lyases are generally classified into three groups, i.e., polyM-specific lyases (EC 4.2.2.3), polyG-specific lyases (EC4.2.2.11), and bifunctional lyases [8]. According to amino acid sequence information, alginate lyases are assigned to PL5, PL6, PL7, PL14, PL15, PL17, PL18, PL32, PL34, and PL36 families in the Carbohydrate-Active enZYmes (CAZy) database (http://www.cazy.org/) [9,10]. Moreover, based on their action modes, alginate lyases are generally classified into endolytic and exolytic enzymes [11]. Endolytic alginate lyases depolymerize alginate into alginate oligomers, and exolytic alginate lyases could digest alginate oligosaccharides by further producing alginate monomers [12]. Thus, alginate lyases, especially endolytic enzymes, are useful molecular scissors to trim alginate into bioactive AOS which has been applied widely in food additives, medicine, and agronomy [13]. To today, various alginate lyases have been isolated from diverse organisms including seaweeds, marine mollusks, marine bacteria, marine fungi, and viruses [14]. However, the commercial exploitation of alginate lyases is largely restricted by lacking suitable enzymes with high activity and adaptive capacity to the industrial environment [15]. Therefore, screening alginate lyases with high catalytic activity and broad environmental suitability is meaningful for utilizing the abundant seaweed polysaccharides.

Agricultural application of AOS has been reported variously. For instance, AOS could promote root growth by regulating the auxin content and auxin signaling in rice (*Oryza sativa* L.) [16]. AOS with lower DP could relieve growth inhibition due to salt stress and alleviate the damage of salt stress by effectively increasing antioxidant enzyme activities e.g., guaiacol peroxidase (POD) and superoxide dismutase (SOD) [17]. Moreover, AOS could enhance wheat (*Triticum aestivum* L.) tolerance to drought and *Arabidopsis thaliana* resistance to Pst DC3000 by regulating the ABA-dependent signaling pathway and salicylic acid-mediated signaling pathway, respectively [18,19]. However, the elicitor-active effect of AOS against chilling has not been elucidated.

In this work, an endolytic polyM-specific alginate lyase, AlgSH7, was purified and characterized from an efficient alginate-degrading bacterium *Microbulbifer* sp. SH-1 which was newly isolated from coastal soil. In addition, to evaluate the potential application of AlgSH7 in agronomy, the degradation products were determined and their elicitor activity against chilling was investigated in Chinese flowering cabbage (*Brassica parachinensis* L.).

## 2. Results

### 2.1. Isolation and Identification of the Effective Alginate-Degrading Bacterium SH-1

**Figure 1:**
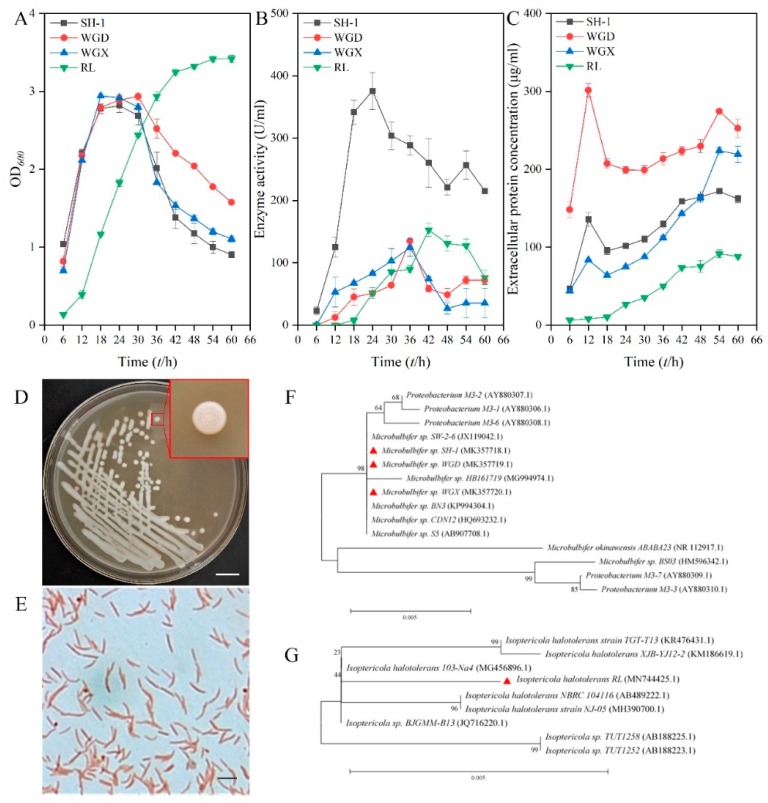
Isolation and identification of the effective alginate-degrading bacterium SH-1. (**A**) Changing of biomass in the culture of four selected strains. (**B**) Changing of enzyme activity in the culture supernatant of four selected strains. (**C**) Changing of proteins within the strain’s supernatant. (**D**) Colony morphology of strain SH-1. Bar = 1cm. (**E**) Cellular morphology of strain SH-1. Bar = 2 μm. (**F**) Neighbor-joining phylogenetic tree derived from the 16S rRNA sequence of strain SH-1, WGD, and WGX. (**G**) Neighbor-joining phylogenetic tree derived from the 16S rRNA sequence of strain RL. Numbers in parentheses represent accession numbers in GenBank. Numbers at each branch point represent the bootstrap values of 1000 trials. Bar 0.005 is the sequence divergence.

Using sodium alginate as the sole carbon source in the medium, 12 strains with clearing zones on the screening plates were screened and isolated from the coastal soil from Zhangzhou, Fujian province, P.R. China. Among them, the fermentation supernatant of four strains, which were numbered as SH-1, WGD, WGX, and RL, respectively, showed obvious alginate lyase activity. As shown in Figure 1F,G, the 16S rRNA genes of strain SH-1 (MK357718.1), WGD (MK357719.1), WGX (MK357720.1), and RL (MN744425.1) were cloned and compared with available 16S rRNA sequences from the nucleotide collection database of GenBank. BLASTn analysis on sequence similarity revealed that strain SH-1, WGX, and WGD were located in the same clade with *Microbulbifer* sp. SW2-6 (JX119042.1) and shared 100%, 99.93%, and 99.86% identities with *Microbulbifer* sp. SW2-6 (JX119042.1), respectively. The results suggested that strain SH-1, WGD, and WGX are members of the genus *Microbulbifer* sp. and designated as *Microbulbifer* sp. SH-1, *Microbulbifer* sp. WGD, and *Microbulbifer* sp. WGX (Figure 1F). Likewise, strain RL is identified as a member of *Isoptericola halotolerans* and named as *Isoptericola halotolerans* RL (Figure 1G).

The growth and enzyme production curve of four strains are shown in Figure 1A,B. Strain SH-1, WGD, and WGX reached to the plateau stage at 18 h, and no significant difference was observed among the biomass of the other three strains. The enzyme activity of strain SH-1 reached up to 375.91 U/mL at 24 h, which was statistically higher than the maximum enzyme activities of strain WGD and WGX. The growth and enzyme production of RL lagged behind strain SH-1, and its maximum enzyme activity, which was 152.57 U/mL after 42 h cultivation, was significantly lower than strain SH-1. However, its biomass was notably higher than strain SH-1 when the plateau stage was reached. The amount of proteins within the strain’s supernatant were also considered. As shown in Figure 1C, although the protein within SH-1’s supernatant was higher than RL’s, the specific activity of SH-1 was still higher than RL when they each reached the maximum enzyme activity. These results indicated that a single cell of strain SH-1 is more efficient in producing alginate lyase. Thus, *Microbulbifer* sp. SH-1 is selected as efficient alginate lyase-producing strain for further study. Morphological analysis showed that colonies of *Microbulbifer* sp. SH-1 are milky white rounds with an orderly brim as shown in Figure 1D. Moreover, strain SH-1 is a rod (approximately 2–3 μm), Gram-negative bacterium as shown in Figure 1E. 

### 2.2. Purification and Identification of the Alginate Lyase AlgSH7

**Figure 2:**
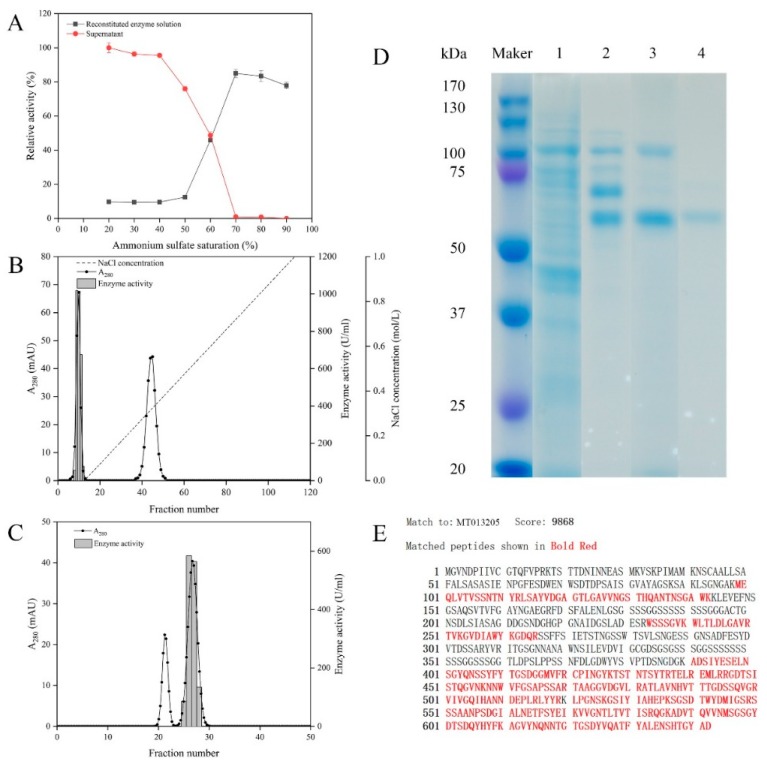
Purification and identification of the alginate lyase AlgSH7. (**A**) Ammonium sulfate precipitation of AlgSH7. (**B**) Fractionation of the AlgSH7 by DEAE-Sepharose Fast Flow column. (**C**) Fractionation of the AlgSH7 by Sephadex G-75. (**D**) SDS-PAGE of AlgSH7 during the purification process. Lanes 1–4: proteins from liquid supernatant, (NH4)_2_SO_4_ fractionation, DEAE-Sepharose Fast Flow, and Sephadex G-75, respectively. (**E**) Identification of purified AlgSH7. Sequence alignment of polypeptide chain fragments (identified by ESI-Q-TOF MS) with the deduced amino acid sequence of AlgSH7. The peptides that match the deduced amino acid sequence of the enzyme are bold red.

*Microbulbifer* sp. SH-1 was cultured in an optimized liquid medium for 24 h until its enzyme activity reached the maximum. Then various steps were performed in the purification process to purify alginate lyase from the fermentation supernatant. Ammonium sulfate precipitation was the first purification step. Figure 2A shows alginate lyase activity of the precipitates and supernatants obtained at different ammonium sulfate saturation processes. Obviously, the relative enzyme activity of precipitates enhanced with the increase of ammonium sulfate saturation. When the ammonium sulfate saturation reached 70%, over 80% of enzyme activity could be recovered. Whereas, the enzyme activity barely continued to rise in the case of saturation beyond 70%. Therefore, ammonium sulfate saturation of 70% was selected to precipitate alginate lyase from the fermentation supernatant of *Microbulbifer* sp. SH-1. The precipitate obtained by 70% ammonium sulfate saturation was purified by an anion-exchange chromatography column and then applied to Sephadex G-75. The elution profile of DEAE-Sepharose Fast Flow chromatography is shown in Figure 2B. Two peaks were eluted at NaCl concentrations of 0 and 0.42 mol/L, respectively. The first peak showed distinct enzyme activity, and the fractions 8-12 with high alginate lyase activity were pooled. The active fractions were then applied to Sephadex G-75. Two protein peaks and a single enzyme activity peak were eluted by gel filtration (Figure 2C). The active fractions were collected, and purified alginate lyase was named AlgSH7. 

Table 1 provides a summary of the alginate lyase purification process. Compared with the crude enzyme, AlgSH7 was purified 7.94-fold, with a recovery yield of 6.60%. The specific activity of purified AlgSH7 reached up to 12,908.26 U/mg. Purified AlgSH7 showed a single band with a molecular weight of approximately 66.0 kDa on sodium dodecyl sulfate polyarylamide gel electrophoresis (SDS-PAGE) followed by Coomassie Blue staining (Figure 2D). Based on gene annotation of the *Microbulbifer* sp. SH-1 genomic sequence, a 1929 bp putative alginate lyase gene (MT013205.1, designated as algsh7), encoding a protein of 642 amino acid residues with a theoretical molecular mass of 66.4 kDa and theoretical pI 4.71, was identified. The molecular weight of purified AlgSH7 (66.0 kDa), which was determined by SDS-PAGE, is consistent with the theoretically predicted molecular weight (66.4 kDa). The purified AlgSH7 was hydrolyzed by trypsin and submitted to ESI-Q-TOF MS. The data were analyzed by using MASCOT search engine (http://www.matrixscience.com), and a C.I% protein score greater than 70 was regarded as significant (*p* < 0.05) [20]. Mascot searching results against GenBank database showed that AlgSH7 match to its theoretical sequence algsh7 from *Microbulbifer* sp. SH-1 with protein scores of 9868, and the protein coverage of purified enzyme is 50% against the theoretical sequence (Figure 2E).

**Figure 3:**
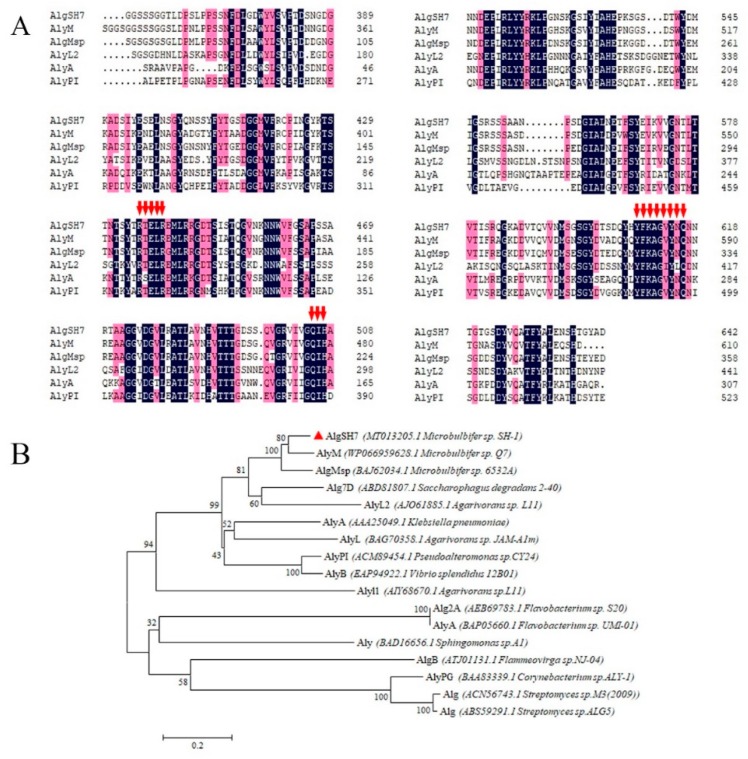
Sequence analysis of AlgSH7. (**A**) Multiple sequences alignments of AlgSH7 and alginate lyases AlgM (WP 066959628.1) from *Microbulbifer* sp. Q7, AlgMsp (BAJ62034.1) from *Microbulbifer* sp. 6532A, AlyL2 (AJO61885.1) from *Agarivorans* sp. L11, AlyA (AAA25049.1) from *Klebsiella pneumoniae* subsp. *Aerogenes* and AlyPI (ACM89454.1) from *Pseudoalteromonas* sp. CY24. The conserved regions are highlighted with red arrows. (**B**) Neighbor-joining phylogenetic tree of AlgSH7 based on putative protein sequences.

Multiple sequences alignments of AlgSH7 and related alginate lyases from the PL7 family are shown in Figure 3A. PL7 family alginate lyases are identified by containing three highly conserved domains, SA3 (RXEXR), SA4 (YXKAGXYXQ), and SA5 (QXH) [21]. Three highly conserved regions RTELR, YFKAGVYNQ, and QIH are identified in the amino acid sequence of AlgSH7, suggesting that AlgSH7 is a new member of the PL7 family. As shown in Figure 3B, the neighbor-joining phylogenetic tree was constructed based on the amino acid sequence of AlgSH7 and available alginate lyases belonged to the PL7 family in the CAZy database (http://www.cazy.org/). In the phylogenetic tree, AlgSH7 is 83.6% homologous with alginate lyase AlgMsp (BAJ62034.1) from Microbulbifer 6532A [22] and 73.22% homologous with alginate lyase AlyM (WP 066959628.1) from Microbulbifer sp. Q7 [23] (Figure 3A).

### 2.3. Biochemical Characterization of AlgSH7

**Figure 4:**
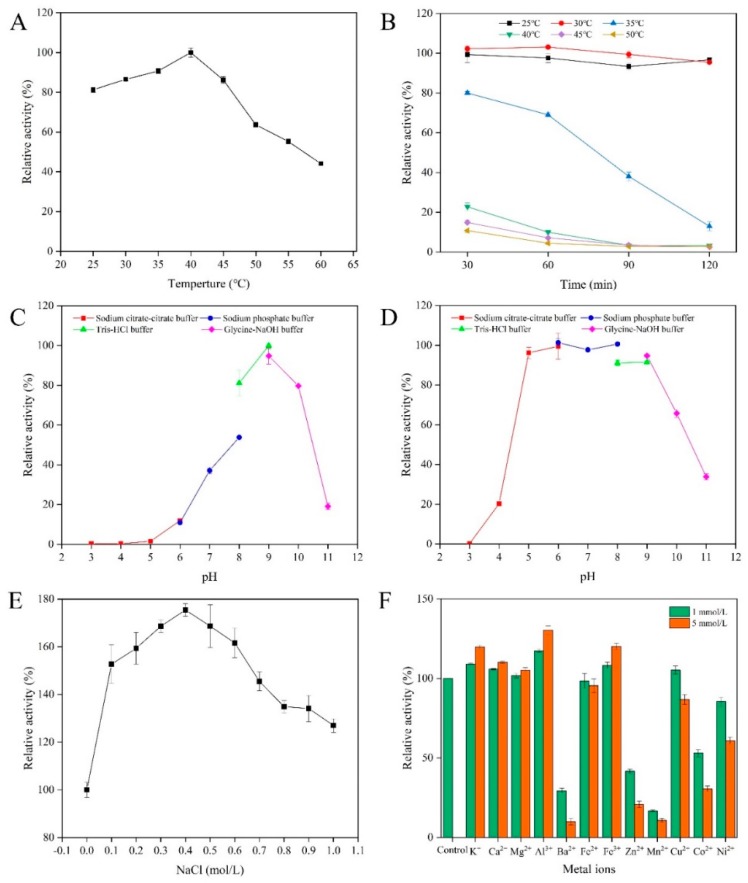
Biochemical characterization of AlgSH7. (**A**) Effect of temperature on AlgSH7 activity. (**B**) Thermostability of AlgSH7. (**C**) Effect of pH on AlgSH7 activity. (**D**) pH stability of AlgSH7. (**E**) Effects of NaCl on AlgSH7 activity. (**F**) Effects of metal ions on AlgSH7 activity. The activity of AlgSH7 without any metal ions in the reaction mixture was taken as the control (100%).

The optimal temperature of AlgSH7 was investigated over the temperature range of 25–60 °C. AlgSH7 exhibits over 80% activity between 25 and 45 °C, and the optimal reaction temperature is 40 °C (Figure 4A). The thermostability of AlgSH7 was determined by measuring the residual activities after incubating the enzyme at 25, 30, 35, 40, 45, and 50 °C for different times, respectively. The enzyme displays over 90% of the maximum enzyme activity after 2-h incubation at 25 and 30 °C. After incubated at 35 °C for 30 min and 2 h, the residual activities are 80% and 13%, respectively. However, AlgSH7 has nearly no activity when the incubation temperature is over 40 °C (Figure 4B). These results indicated that AlgSH7 is sensitive to thermal stress.

The optimal pH of AlgSH7 was investigated at various pH (3.0–11.0) at 40 °C. AlgSH7 displays the highest activity at pH 9.0 and exhibits more than 80% activity in the range of pH 8.0–10.0. There is no detectable enzyme activity at pH 3.0–5.0 (Figure 4C). The pH stability was evaluated by determining the residual activities after treating the enzyme with different pH buffer (3.0–11.0) at 4 °C for 24 h. The results showed that AlgSH7 remains stable over a broad pH range of 5.0–9.0 and maintains approximately 90% of the enzyme activity. The AlgSH7 loses more than 40% of its activity when the pH is below 4.0 or above 10.0 (Figure 4D). These results implied that AlgSH7 is an alkaline enzyme, and had excellent pH stability.

The effects of NaCl concentrations on the enzyme activity of AlgSH7 are shown in Figure 4E. NaCl concentrations at a range of 0.1 to 1 mol/L promote the enzyme activity of AlgSH7, and the optimal concentration for AlgSH7 activity is 0.4 mol/L, which boosts approximately 75% compared to the control without NaCl. Thus, AlgSH7 is a salt-activated enzyme. The effects of metal ions on AlgSH7 activity were determined by adding various metal ions at concentrations of 1 and 5 mmol/L. Metal ions including K^+^, Ca^2+^, Mg^2+^, Al^3+^, and Fe^3+^ display activating effects at 1 and 5 mmol/L, and the stimulation effect is much more obvious at a high concentration. The trivalent metal ion Al^3+^ displays the most promoting effect with 130.25% of relative activity followed by Fe^3+^ with 120.12%. Instead, some cations, such as Ba^2+^, Zn^2+^, Mn^2+^, Co^2+^, and Ni^2+^, show significant inhibitory effects. It is noteworthy that Cu^2+^ promotes the enzyme activity at a low concentration (1 mmol/L) and inhibits it at a high concentration (5 mmol/L) (Figure 4F). These results indicated that AlgSH7 has an extensive resistance to metal ions, allowing it to display high activity in the environment containing various ions.

### 2.4. The Substrate Specificity of AlgSH7

**Figure 5:**
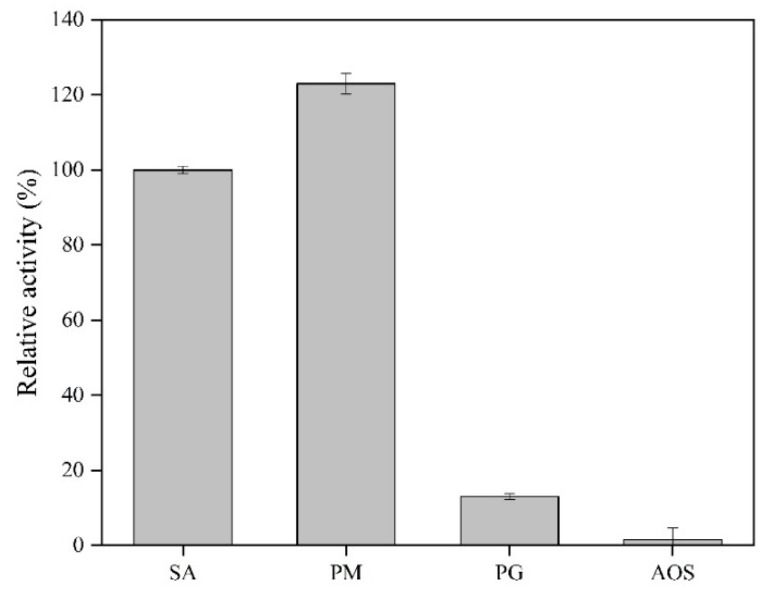
Substrate specificity of AlgSH7. The activity towards sodium alginate was determined as the 100% relative activity.

As shown in Figure 5, AlgSH7 is active in degrading polyM and alginate, and exhibits a very low activity towards polyG, implying that AlgSH7 prefers polyM to polyG. Moreover, AlgSH7 has no catalytic activity towards alginate oligosaccharides. These results indicated that the substrate specificity of AlgSH7 is polyM-specific, and the reaction pattern of AlgSH7 may be endolytic mode. The substrate specificities of PL7 alginate lyases are related to the amino acid sequence in the conserved regions, which formed the cavity structure to bind to suitable substrates [24]. Recent studies have declared that polyM-specific, polyG-specific, and polyMG-specific alginate lyases contained QVH, QIH, and QIH in the conserved regions, respectively [8]. However, AlgSH7 is contrary to this accepted rule. AlgSH7 is active towards polyM, containing the QIH sequence which is similar to AlyA-OU02 [24], AlyPM [25], and FlAlyA [26].

### 2.5. Analysis of the Hydrolysis Products

**Figure 6:**
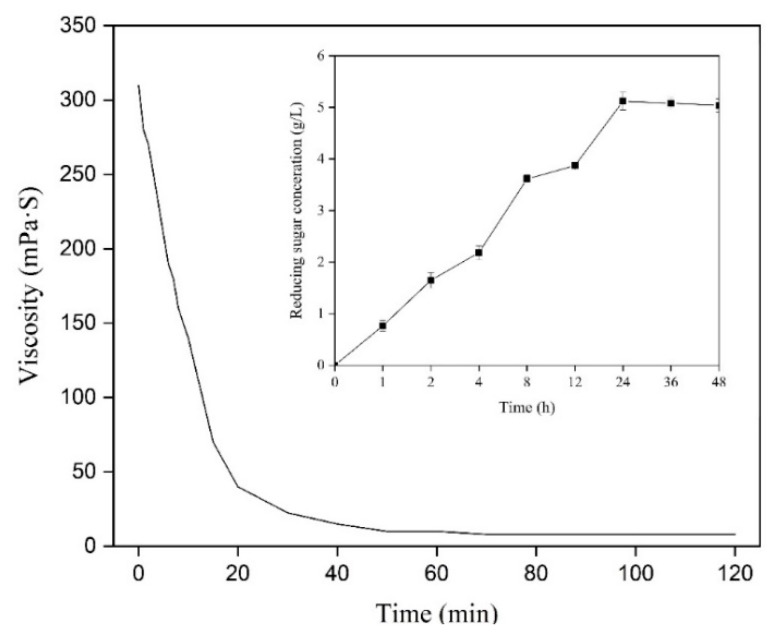
Changes in viscosity and reducing sugars at various time points during AlgSH7 degradation reaction.

The degradation effect of AlgSH7 towards 1% (*w*/*v*) alginate substrate was studied by adding 1% (*v*/*v*) purified enzyme. During the reaction, the viscosity of alginate sharply decreased from 310 to 40 mPa·S within the initial 20 min and finally dropped to 10 mPa·S after 60 min. Meanwhile, an initial increase in the level of reducing sugars was investigated, followed by a continuous rise after 60 min. The concentration of reducing sugar reached a maximum at 24 h and then remained constant (Figure 6). These results implied that AlgSH7 can rapidly degrade the viscosity-producing alginate macromolecules into small molecular blocks with no viscosity. With further degradation, those small blocks continue to depolymerize until the endpoint is reached. Therefore, the products degraded by AlgSH7 for 24 h were selected as end products for subsequent analysis.

**Figure 7:**
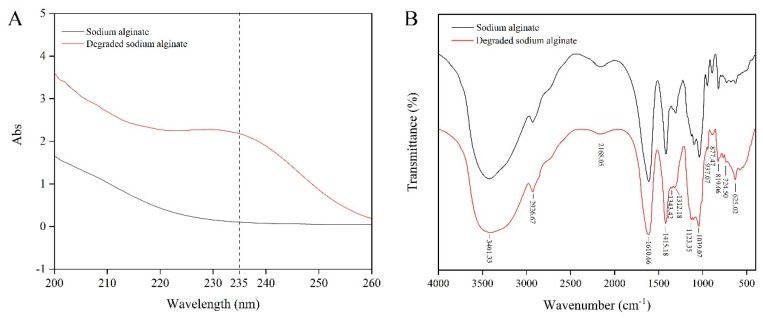
UV and FT-IR analyses of alginate and the AlgSH7 hydrolysis products. (**A**) The UV scan spectrogram from 200 to 260 nm of sodium alginate and its hydrolysis products. (**B**) FT-IR spectrogram of sodium alginate and its hydrolysis products.

The properties of alginate and its degradation products were compared using UV and FT-IR spectroscopy. The UV scanning results of sodium alginate and its hydrolysis products show a common characteristic absorption peak of sugars at 200 nm. In addition, the hydrolysis products exhibit a unique peak at a wavelength of 235 nm, which is a clear indication of the production of unsaturated oligosaccharides [27], indicating a significant increase in the level of unsaturated sugars in degraded alginate (Figure 7A). The data from FT-IR analyses found that alginate and the AlgSH7 hydrolysis products have basically identical characteristic peaks, indicating that hydrolysis of alginate does not lead to significant side group changes. In the FT-IR spectrum, the wide peak at 3401 cm^−1^ is assigned to O–H stretching vibration. The peak at 2926 cm^−1^ is attributed to a C-H stretching vibration. The peaks at 1610 and 1415 cm^−1^ are asymmetrical O–C–O and symmetrical O–C–O stretching vibrations, respectively. The peaks at 1343 and 1312 cm^−1^ are correlated with the M and G content, respectively [23] (Figure 7B).

**Figure 8:**
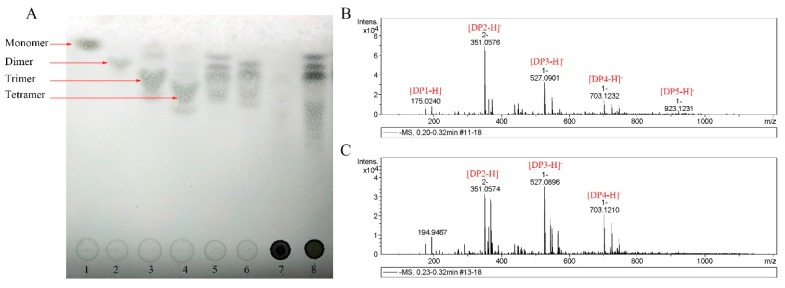
TLC and ESI-MS analysis of the hydrolysis products. (**A**) TLC analysis of the hydrolysis products of sodium alginate. Lanes 1–4: monomer, dimer, trimer, tetramer, respectively; Lanes 5–7: hydrolysis products of 0.5% sodium alginate, 0.5% polyM, and 0.5% polyG, respectively; Lane 8: hydrolysis products of 1% sodium alginate. (**B**) ESI-MS analysis of the degradation products of AlgSH7 with alginate as substrate. (**C**) ESI-MS analysis of the degradation products of AlgSH7 with the polyM as substrate. The data highlighted in red represent the relative abundance of peaks.

The hydrolysis products of sodium alginate were analyzed by thin-layer chromatographic (TLC) to investigate the reaction modes of AlgSH7. As shown in Figure 8A, the bands of disaccharides, trisaccharides, and tetrasaccharides are clearly observed on the lanes of hydrolysis products of sodium alginate and polyM, and no bands appear on the lane of hydrolysis products of polyG (Figure 8A). The results indicated that AlgSH7 is a strict polyM-specific alginate lyase. To confirm the results analyzed by TLC, hydrolysis products were further analyzed by ESI-MS. As shown in Figure 8B,C, under the negative mode, the ion peaks at 351, 527, and 703 m/z represent unsaturated disaccharides ([DP2 − H]^−^), trisaccharides ([DP3 − H]^−^), and tetrasaccharides ([DP4 − H]^−^), respectively. The hydrolysis products of alginate (Figure 8B) and polyM (Figure 8C) all contain these peaks, which is consistent with the result of TLC. Thus, AlgSH7 mainly acts on the substrate in an endolytic pattern.

### 2.6. Eliciting Activity of Oligosaccharides against Chilling in Chinese Flowering Cabbage

**Figure 9:**
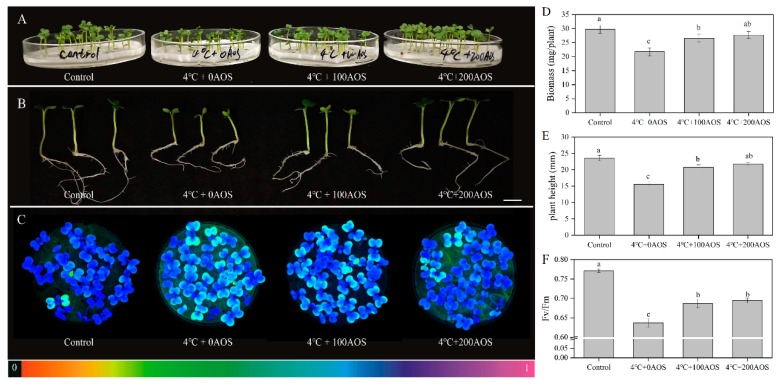
Effects of alginate oligosaccharides on Chinese flowering cabbage under chilling stress. (**A**) Effects of alginate oligosaccharides (AOS) on phenotypes of Chinese flowering cabbage seedlings in plate under chilling stress. (**B**) Effects of AOS on individual phenotypes of Chinese flowering cabbage seedlings, bar = 1 cm. (**C**) The maximum quantum yield of PS II (Fv/Fm). The underneath false color code depicted in the image, ranges from 0 (black) to 1 (purple). (**D**–**F**) Effects of AOS on biomass (**D**), plant height (**E**), and Fv/Fm value (**F**) under chilling stress.

The low temperature is one of the major adverse environmental stresses posing damage to horticultural crops and causing huge yield loss [28]. The application of functional chemical compounds could be a vital way to enhance plant abiotic resistance. AOS prepared from the degradation of alginate is a potent plant elicitor. To explore the effect of hydrolysis products prepared by AlgSH7 on chilling resistance in Chinese flowering cabbage (*Brassica parachinensis* L.cv.biqing). The biomass, plant height, and the maximum quantum yield of PS II (Fv/Fm) of AOS-pretreated and non-AOS-pretreated seedlings under chilling stress were compared (Figure 9). After 3-d chilling stress (4 °C) treatment, the growth of seedlings without AOS pretreatment was significantly inhibited, leading to 33.89% and 26.86% decrease of biomass and plant height, respectively. However, 200 mg/L AOS pretreatment statistically increased biomass and plant height by 27.36% and 39.74% compared with the seedlings without AOS pretreatment. A similar effect was also observed in 100 mg/L AOS pretreatment. (Figure 9D,E). The maximum quantum yield of PS II (Fv/Fm) reflected the photosynthetic performance of plants and was generally used as a vital indicator to judge the degree of environmental stress on plants. As depicted in Figure 9C,F, purple-blue color represents the normal state of photosynthetic performance, whereas green or yellow colors indicated damage of photosystem II induced by chilling. Chilling (4 °C) treatment dramatically decreased Fv/Fm in seedlings without AOS pretreatment. It was notable that significantly higher Fv/Fm was observed in AOS-pretreated seedlings than in non-AOS-pretreated ones (Figure 9F). These results implied that the exogenous application of AOS could enhance the tolerance of Chinese flowering cabbage to chilling.

## 3. Discussion

In this study, an efficient alginate-degrading bacterium *Microbulbifer* sp. SH-1 was isolated from coastal soil. The *Microbulbifer* genus was originally proposed by González and typically found in marine sediments, salt marshes, coastal soil, and mangroves [29]. Reported bacterial strains of the *Microbulbifer* genus can efficiently degrade multiple marine polysaccharides, such as alginate, agarose, and carrageenan, implying the *Microbulbifer* genus may be a potential resource for various efficient polysaccharide-degrading enzymes [30,31]. However, to date, only four alginate lyases, which are ALW1 and AlgL17 (KY780301) from *Microbulbifer* sp. ALW1 [32,33], AlgMsp (BAJ62034.1) from *Microbulbifer* 6532A [22], and AlyM (WP066959628.1) from *Microbulbifer* sp. Q7 [23], have been isolated and characterized from the *Microbulbifer* genus. Herein, an extracellular alginate lyase, AlgSH7, from *Microbulbifer* sp. SH-1 was purified and characterized. Protein sequence analysis indicates the sequence of purified AlgSH7 match to algsh7 (MT013205), which is one of three putative alginate lyase genes identified from the *Microbulbifer* sp. SH-1 genomic sequence (CP046948). AlgSH7 contains 642 amino acids with a theoretical molecular mass of 66.4 kDa and theoretical pI 4.71. AlgSH7 belongs to the PL7 family because it contains the SA3 domain (RTELR), the SA4 domain (YFKAGVYNQ), and the SA5 catalytic domain (QIH), which are conserved in the aligned catalytic modules of PL7 family alginate lyase [21,25]. Among the characterized alginate lyases derived from the *Microbulbifer* genus, AlgSH7 is 83.6% homologous with alginate lyase AlgMsp (BAJ62034.1) and 73.22% homologous with alginate lyase AlyM. In alginate degradation, AlgSH7 displays polyM-specific property, while, AlyM is a polyG-specific alginate lyase, and AlgMsp is a bifunctional alginate lyase. Moreover, the molecular weight of AlgSH7 (66.4 kDa) is higher than AlgMsp (37.0 kDa) and AlyM (62.9 kDa). These results indicated that AlgSH7 is a new PL7 family enzyme from the *Microbulbifer* genus.

So far, based on the CAZy database (http://www.cazy.org/), more than 1000 gene sequences and at least 38 characterized proteins belonging to the PL7 family have been found in bacteria. Alginate lyases vary in substrate specificities, and they are commonly assigned to G-specific, M-specific, and bifunctional groups [11]. The conserved amino acids play vital roles in catalyzing reaction or forming jelly roll β-sandwich structure, which is regarded as a binding site of a suitable substrate [24,34]. It has been reported that the conserved regions of polyM-preference, polyG- preference, and polyMG-preference alginate lyases contain QVH, QIH, and QIH amino acid residues, respectively [24]. For example, AlgNJU-03, AlgM4, and AlyPI contain QIH in the conserved region and show activities towards polyM and polyG [21,34,35]. The polyG-preference alginate lyases, such as ALG-5, AlyM, and Alg2A all contain an QIH sequence [23,36,37]. For polyM-specific alginate lyases, A9mT and PyAly all contain QVH [38,39]. However, AlgSH7 is contrary to this accepted rule. AlgSH7 contains an QIH sequence, while, it prefers polyM blocks. This result is similar to AlyA-OU02, AlyPM, and FlAlyA [24,25,26]. Most of the reported PL7 family alginate lyases have endolytic activity. Alginate lyases with endolytic activity generally act on glycosidic bonds within the linear polysaccharides chain of alginate, releasing unsaturated alginate oligomers which are dominated by disaccharides, trisaccharides, and tetrasaccharides [40]. Exolyases further depolymerize these oligosaccharides into monosaccharides [5]. The degradation products of alginate and polyM prepared by AlgSH7 mainly consist of oligosaccharides with DP of 2–4, which is similar to other polyM-specific enzymes in the PL7 family, such as AlgA from *Bacillus* sp. Alg07 [6], FlAlyA from *Flavobacterium* sp UMI-01 [26], and AlgNJ-07 from *Serratia marcescens* NJ-07 [15]. Thus, AlgSH7 mainly acts on the substrate in an endolytic mode. In addition, AlgSH7 displays no activity on oligosaccharides, confirming that AlgSH7 is an endolytic enzyme. Therefore, AlgSH7 displays a potential application for producing lower molecular weight guluronate-enriched alginate. 

Several cold-adapted alginate lyases have been reported, such as AlyPM, TsAly6A, and AlyL1 [25,41,42]. Compared with mesophilic alginate lyases, which usually have optimal temperatures around 50 °C and are stable at temperatures lower than 50 °C [25], cold-adapted alginate lyases usually have lower optimal temperatures, higher activity at low temperatures, and lower thermostability [42]. Consistent with these features, the optimal reaction temperature of AlgSH7 is 40 °C, and AlgSH7 shows over 80% of the maximum activity over 80% at 25 °C. Moreover, AlgSH7 is unstable at temperatures beyond 30 °C. These results indicated that AlgSH7 is a cold-adapted alginate lyase. The cold-adapted property allows the catalytic reaction to be easily started and terminated by controlling the temperature slightly. Compared with most reported alginate lyases which have optimal activity in the range of pH 6.0–8.0 [8,27]. AlgSH7 prefers to show high enzyme activity in alkaline environments, suggesting AlgSH7 is an alkaline alginate lyase. Pretreatment with alkaline hydrolysis is a commonly used method for improving enzymatic saccharification of biomass [43]. The alkaline-preference property of AlgSH7 allows it to be efficiently applied to the industrial production of oligosaccharides.

Alginate lyases can be affected by various metal ions [21]. Na^+^ functions as an activator for many alginate lyases. For instance, 0.2 mol/L NaCl can effectively increase the activity of AlyA-OU02 from *Vibrio splendidus* OU02 [24]. AlyH1 from *Vibrio furnissii* H1 has the highest activity in the presence of 0.3 mol/L NaCl [44]. A similar phenomenon is observed with AlgSH7. In the presence of 0.4 mol/L NaCl, the enzyme activity of AlgSH7 is increased by 1.75-fold, suggesting AlgSH7 is a salt-activated enzyme. The enhancing effect of Na^+^ may due to altering the secondary structure of AlgSH7, which enhances the affinity of the enzyme for its substrates and facilitates enzymolysis [21,25]. The activation effects of metal ions on alginate lyases, such as K^+^, Ca^2+^, and Mg^2+^, have been widely reported [45]. These ions are believed to play vital roles in protecting enzyme structure and stimulating catalytic activity [12]. Consistent with these reports, metal ions including K^+^, Ca^2+^, and Mg^2+^ display activating effects at 1 and 5 mmol/L, and the stimulation effects are much more obvious at a high concentration. It is notable that trivalent metal ions Al^3+^ and Fe^3+^, which generally have a destructive effect on enzyme activity, such as AlySY08 from *Vibrio* sp. SY08 [46] and AlgA from *Bacillus* sp. Alg07 [6], display the most promoting effects with 130.25% and 120.12% of relative enzyme activity. To our knowledge, this is the first report that the AlgSH7 shows the above properties. AlgSH7 can be activated by many metal ions, especially Na^+^, Al^3+^, and Fe^3+^. These properties enable it to maintain high activity in the complex environment containing various ions.

Low temperature is one of the major adverse environmental stresses posing damage to horticultural crops and causing huge yield losses [47]. The application of functional chemical compounds is a vital way to enhance plant abiotic resistance. AOS as the degradation product of alginate, especially enzymatic hydrolysis products of alginate, is a potent plant elicitor [16]. Previous studies have reported that alginate oligosaccharides are effective in stimulating seed germination [48], promoting root growth [16], enhancing plant tolerance to drought and salt stress [18,49], and alleviating toxic effects of Cd [50]. However, the elicitor-active effect of degradation products against chilling has not been thoroughly elucidated. Our data from Figure 9 indicates that AlgSH7 hydrolysis products notably promote the growth of Chinese flowering cabbage under chilling stress. Meanwhile, the photoinhibition caused by chilling can be relieved by the oligosaccharides produced by AlgSH7. These results are consistent with the previous research that exogenous chitooligosaccharides (COS) treatment increased plant height and fresh weight in rice, and foliar-applied oligocarrageenan improved photosynthetic parameters in peppermint under chilling stress [51,52]. A recent study reports that pretreatment with COS can induce the expression of PSII D1 protein-encoding genes, indicating it plays an important role in the repairing process of photo damaged PSII [28]. The alleviating effect of alginate oligosaccharides on the cold stress-induced damage to photosystem II may due to the same mechanism. Higher PSII activity in AOS-pretreated seedlings maintain stronger photosynthesis in comparison with non-AOS-pretreated ones, which show better seedlings growth under chilling stress. These results suggest that oligosaccharides derived from the enzymatic hydrolysis of alginate can help plants to overcome chilling stress in agriculture.

## 4. Materials and Methods 

### 4.1. Materials

Coastal soil was collected from Zhangzhou (24.15 N 117.95 E), Fujian Province, P.R. China. Sodium alginate (1% aqueous solution, 300 mPa·S, purity: >99%) from brown algae was purchased from Sigma (St. Louis, MO, USA). PolyM (purity: >99%), polyG (purity: >99%) and commodity alginate oligosaccharides (purity: >85%) were purchased from Qingdao BZ Oligo Biotech Co., Ltd. (Qingdao, China). DEAE-Sepharose Fast Flow column and Sephadex G-75 were purchased from REBIO Co., Ltd. (Shanghai, China).

### 4.2. Screening and Isolation of Alginate-Degrading Strains

The 5 g sample of coastal soil was added to 95 mL sterilized water, and then 1 mL mixture was transferred to 50 mL enrichment medium (%, *w*/*v*) containing 1% sodium alginate, 1.5% NaCl, 0.5% (NH_4_)_2_SO_4_, 0.1% K_2_HPO_4_, 0.1% MgSO_4_·7H_2_O, and 0.001% FeSO_4_·7H_2_O (pH 7.5). After cultivated at 30 °C for 48 h, microorganisms that could grow in enrichment medium were spread on sodium alginate-agar plates for isolation. The plates were incubated at 30 °C for 48 h and then poured with 10% (*w*/*v*) cetyl pyridine chloride (CPC). The colonies showing clear zones were separated and transferred to new plates to obtain pure cultures. Subsequently, the pure strains were transferred to the liquid medium and incubated aerobically with the same conditions as the above for rescreening strains with high alginate lyase activity. Enzyme activity was determined using the procedures described below.

### 4.3. Identification of Microbulbifer sp. SH-1

The bacterial strain *Microbulbifer* sp. SH-1 (CGMCC No.16906) was identified based on its morphological features and 16S rRNA gene sequence analysis. Light microscopy (Nikon Eclipse E600, Japan) with a 100× oil immersion lens was used for morphological observation. The 16S rRNA gene of the strain was amplified through PCR by using universal primers. The amplification product was purified, then directly sequenced by Sangon Biotech (Shanghai, China). BLASTn (https://blast.ncbi.nlm.nih.gov/) was used for comparing purified PCR fragment sequences with reported 16s rRNA sequences in Genbank (https://www.ncbi.nlm.nih.gov/genbank/). A phylogenetic tree was constructed using MEGA 6.0 (https://www.megasoftware.net/) through the neighbor-joining method [53].

### 4.4. Crude Enzyme Collection and Ammonium Sulfate Fractionation 

Strain *Microbulbifer* sp. SH-1 was cultured in 1 L optimized liquid medium (%, *w*/*v*) containing 1% sodium alginate, 0.5% NaCl, 0.5% (NH_4_)_2_SO_4_, 0.1% K_2_HPO_4_, 0.02% MgSO_4_·7H_2_O, and 0.002% FeSO_4_·7H_2_O (pH 7.5) at 32 °C with shaking at 240 r/min for 24 h. Cells of *Microbulbifer* sp. SH-1 were removed by centrifuging at 12,000× *g*, 4 °C for 15 min, and the supernatant was used as a crude enzyme for ammonium sulfate fractionation. According to the ammonium sulfate saturation table (0 °C), ammonium sulfate with various weights was added to the supernatant for protein precipitation. After being placed for 12 h at 4 °C, the sample was centrifuged at 12,000× *g*, 4 °C for 15 min. Then the precipitated protein was redissolved in 20 mmol/L sodium phosphate buffer (PB, pH 7.5) of an equal volume to the supernatant. Finally, the activities of enzymes that were obtained from different ammonium sulfate saturation fractionations were assayed as described below. The optimal saturation was selected for further purification based on the relationship between ammonium sulfate saturation and relative enzyme activity [20].

### 4.5. Purification of Alginate Lyase 

The enzyme solution from the 70% ammonium sulfate fractionation was dialyzed overnight against a large volume (5 L) of PB buffer (pH 7.5) to remove the residual ammonium sulfate. It was then subjected to AKTA FPLC (GE Healthcare Life Science, Marlborough, MA, USA) equipped with a DEAE-Sepharose Fast Flow column (2.6 × 20 cm, REBIO, Shanghai, China), which had been desalted and equilibrated with 20 mmol/L PB (pH 7.5). Adsorbed proteins were then eluted with a linear gradient of 0–1.0 mol/L NaCl in PB buffer at a flow rate of 1.0 mL/min. Then, fractions of 4.0 mL each were collected and screened for alginate lyase activity. The fractions with alginate lyase activity were concentrated using a Millipore centrifugal filter 10 K device (Millipore, USA). Then, the sample was further purified by gel filtration on a Sephadex G-75 column (1.6 × 60 cm, REBIO, Shanghai, China), pre-equilibrated with 20 mmol/L PB (pH 7.5). The fractions were eluted with the same buffer at a flow rate of 0.5 mL/min. The active fraction was concentrated and used as a purified enzyme preparation throughout this study [32].

### 4.6. Protein Concentration and Alginate Lyase Activity Assay

The protein concentrations were determined by the Bradford method using bovine serum albumin as the standard [54]. Alginate lyase activity was determined by 3, 5-dinitrosalicylic acid (DNS) colorimetry [55]. Briefly, enzyme solution (0.1 mL) was mixed with 0.9 mL of 0.8% sodium alginate (dissolved in 20 mmol/L PB, pH 7.5) and incubated at 40 °C for 10 min. The reaction was stopped by adding 0.5 mL of DNS reagent and heating at 100 °C for 5 min. After cooling to room temperature, the concentration of reducing sugar was monitored at 540 nm using a UV-2550 spectrophotometer (SHIMADZU, Japan). One unit (U) was defined as the amount of enzyme required to release 1 μg of reducing sugar (glucose equivalent) per min.

### 4.7. Electrophoretic Analysis

The molecular weight of proteins in the purification process was estimated using 12.5% sodium dodecyl sulfate-polyacrylamide gel electrophoresis (SDS-PAGE) which was in accordance with the method of Laemmli [56].

### 4.8. Identification of Purified AlgSH7 

The purified protein bands obtained in SDS-PAGE were excised from the gel and subjected to the in-gel trypsin digestion procedure. For peptide identification, the in-gel digested peptides were analyzed by electrospray ionization quadrupole time-of-flight mass spectrometry (ESI-Q-TOF MS) (Q exactive, Thermo Scientific Co., Waltham, MA, USA). Data acquisitions of the spectra were analyzed using the MASCOT (Matrix Science, Inc., Boston, MA) which was in accordance with the method of Zhu [20]. The whole-genome sequences of *Microbulbifer* sp. SH-1 (CGMCC No.16906) were deposited at DDBJ/EMBL/GenBank under the accession number of CP046948. Three coding genes for alginate lyases were identified after the annotation analysis of genomic sequencing data. Among these genes, AlgSH7 (MT013205) was matched to the above searching result. The theoretical molecular and isoelectric point (pI) of AlgSH7 was computed using the ExPASy Compute pI/Mw tool (https://web.expasy.org/compute_pi/). A neighbor-joining phylogenetic tree was constructed using MEGA 6.0 (https://www.megasoftware.net/) based on alginate lyase protein sequences of PL7 family. The DNAman 6.0 software (https://www.lynnon.com/qa.htmL) was used to obtain multiple sequence alignment [57].

### 4.9. Characterization of Alginate Lyase AlgSH7

The optimal temperature of AlgSH7 was measured by determining its activity at a range of 25–60 °C in 20 mmol/L of PB (pH 7.5). The thermostability was examined with pre-incubation of AlgSH7 at 25, 30, 35, 40, 45, and 50 °C for 0.5, 1, 1.5, and 2 h, individually. Then, the residual activity was tested at 40 °C in 20 mmol/L of PB (pH 7.5). The activity of the enzyme stored at 4 °C was used to represent 100% enzyme activity. The influence of pH on AlgSH7 was determined by assaying enzyme activity in different buffer systems over a broad pH range of 3.0–11.0 at 40 °C. The buffers used were 20 mmol/L acetate buffer (pH 3.0–6.0), 20 mmol/L PB (pH 6.0–8.0), 20 mmol/L Tris-HCl buffer (pH8.0–9.0), and 20 mmol/L glycine-NaOH buffer (pH 9.0–11.0). The pH stability was examined by determining the residual enzyme activity at pH 9.0 and 40 °C after conserving the enzyme in different pH buffers at 4 °C for 24 h. The activity of the enzyme without the treatment was defined as 100%. To investigate the effect of NaCl on enzyme activity, the reaction mixture was supplied with 0 to 1 mol/L NaCl, and then the enzyme activity was assayed. The activity without NaCl was defined as 100%. In the absence of NaCl, 1 or 5 mM of KCl, CaCl_2_, MgCl_2_, AlCl_3_, BaCl_2_, FeSO_4_, FeCl_3_, ZnSO_4_, MnCl_2_, CuCl_2_, CoCl_2_, and NiSO_4_ were added to the reaction systems to test the effects of metal ions on enzyme activity. The reaction system without metal ions was used as the control, and the activity was regarded as 100%. Sodium alginate, polyM, polyG, and commodity AOS at 5 mg/mL each were used to analyze the substrate specificity of AlgSH7. The activity towards sodium alginate was regarded as 100%.

### 4.10. Determination of Viscosity and Reducing Sugar Concentration 

First, 100 mL of 1% sodium alginate (dissolved in 20 mmol/L Tris-HCl buffer, pH 9.0) was mixed with 1 mL of purified enzyme. Then, the viscosity of the mixture was measured using a NDJ-1 rotary viscometer (Jingke Instrument, Shanghai China) at 25 °C. Reducing sugar concentration was determined through 3,5-dinitrosalicylic acid (DNS) colorimetry using glucose as the standard [55].

### 4.11. UV and FT-IR Spectroscopy 

UV spectroscopy of sodium alginate and degraded sodium alginate was carried out at room temperature using an UV-2550 spectrophotometer (SHIMADZU, Kyoto, Japan) in the region of 200–260 nm. Fourier transform-infrared spectrophotometer of sodium alginate and degraded sodium alginate was recorded using PerkinElmer Vertex 70 spectrometer (Bruker, Karlsruhe, Germany). Dried samples were dispersed in KBr pellet and values were expressed in cm^−1^. All the spectra were recorded in absorbance mode in the 4000-400 cm^−1^ regions. Baseline correction and normalization were performed for all the spectra [23].

### 4.12. TLC and ESI-MS Analysis of the Hydrolysis Products

TLC and ESI-MS were applied to analyze the degradation products of sodium alginate and polyM produced by purified AlgSH7. First, 100 mL of 1% sodium alginate (dissolved in 20 mmol/L Tris-HCl buffer, pH 9.0) was mixed with 1 mL purified enzyme and incubated at 40 °C for 24 h. A 5 μL aliquot of the reaction products was subjected to TLC plates (TLC Silica gel 60, Merck) using a solvent system of 1-butanol: acetic acid: water (3:2:2, *v*/*v*) [14]. Bands in TLC plates were visualized by heating at 120 °C for 5 min after spraying with 10% (*v*/*v*) sulfuric acid in ethanol. To further confirm the composition of the products, the equipment UPLC1290-6540B Q-TOF (Agilent Co., Santa Clara, CA, USA) was used. The 2 μL of the sample was introduced by direct infusion into the electrospray ionization source-mass spectrometry (ESI-MS), and the datasets were collected. The oligosaccharides were detected in a negative-ion mode using the following settings: capillary voltage, 3.5 kV; nebulizer, 0.3 bar; dry heater, 180 °C; charging voltage, 2 kV; dry gas, 4.0 L/min; and scanning the mass range, 50–1200 m/z [58].

### 4.13. Elicitor Activity of Oligosaccharides against Chilling in Chinese Flowering Cabbage 

Chinese flowering cabbage (*Brassica parachinensis* L. cv. Biqing) seeds were sterilized using 75% alcohol (*v*/*v*) for 15 min and subsequently triple-rinsed in sterile distilled water. Then, 30 seeds were placed on a filter paper spread at the bottom of a 9 × 9 cm Petri dish, following by germinating at 25 °C in the dark for 24 h. Twelve dishes were prepared in this study and cultured in a growth chamber with a 16/8 h light/dark cycle, at 25/20 °C, respectively, for 4 days. Dishes were randomly divided into four groups and grown in the Hoagland solutions supplied with 0, 0, 100, 200 mg/L AOS produced by AlgSH7. After 3-d treatment, one group without AOS treatment was continually cultured under normal conditions, and the other three groups were treated at 4 °C for 3-d chilling stress. To measure the maximum quantum yield of PS II, an imaging-PAM chlorophyll fluorimeter (IMAG-MAXI; Heinz Walz, Effeltrich, Germany) equipped with a computer-operated PAM-control unit was employed. Seedlings in the dishes were dark-adapted for 30 min, and the minimal fluorescence (Fo) was determined. Then, the seedlings underwent a saturating pulse, and the maximal fluorescence (Fm) was obtained. According to Fm and Fo, the maximum quantum yield of PS II can be calculated as Fv/Fm = (fm − fo)/Fm [59].

### 4.14. Statistical Analysis

All experimental indices were developed under natural conditions and replicated three times. Data was performed analysis of variance using SPSS 19.0 (IBM, Armonk, New York, USA). Sample variability was expressed as means ± standard deviations. The statistical differences in the experiment were determined using Duncan’s multiple range test (*p* < 0.05). 

## 5. Conclusions

In conclusion, AlgSH7, the alginate lyase derived from a newly isolated alginate lyase-producing strain *Microbulbifer* SH-1, is characterized as a novel endolytic polyM-specific alginate lyase belonging to PL7 family. AlgSH7 has cold-adapted, pH-stable, salt-activated, and various metal ions-resistance properties. AlgSH7 can release di-, tri-, and tetrasaccharides from alginate and polyM. The hydrolysis products produced by AlgSH7 could significantly relieve the damage triggered by chilling stress on the leaves of Chinese flowering cabbage. All these properties make AlgSH7 a promising candidate for commercial applications in producing bioactive oligomers, which has potential applications in agriculture to deal with diverse stresses.

## Figures and Tables

**Table 1:** Summary of the purification of AlgSH-1.

Purification Steps	Total Protein (mg)	Total Activity ^1^ (U)	Specific Activity ^2^ (U/mg)	Yield (%)	Purification (Fold)
Crude enzyme	22.87	37,188.00	1626.06	100.00	1.00
(NH_4_)_2_SO_4_ Fractionation	10.06	26,931.27	2677.06	72.42	1.65
DEAE-Fast Flow	1.06	8234.42	7768.32	22.14	4.78
Sephadex G-75	0.19	2452.57	12,908.26	6.60	7.94

^1^ One unit (U) was defined as the amount of enzyme required to release 1 μg of reducing sugar per min. ^2^ Specific activity = total activity/total protein.
